# Protective effects of melatonin on testis histology following acute torsion-detorsion in rats

**Published:** 2017-03

**Authors:** Mehri Mirhoseini, Fereshteh Talebpour Amiri, Abbas Ali Karimpour Malekshah, Zahra Rezanejad Gatabi, Elmira Ghaffari

**Affiliations:** 1 *Amol Faculty of Nursing and Midwifery, Mazandaran University of Medical Sciences, Sari, Iran.*; 2 *Department of Anatomical Sciences, Immunogenetics Research Center, Faculty of Medicine, Mazandaran University of Medical Sciences, Sari, Iran.*; 3 *Department of Pharmaceutics, Faculty of Pharmacy, Mazandaran University of Medical Sciences, Sari, Iran.*

**Keywords:** Spermatic cord torsion, Testis, Melatonin, MDA, Rats

## Abstract

**Background:**

Testicular damage due to spermatic cord torsion may lead to infertility. It is probably because of changes in oxidative stress factors such as malondialdehyde.

**Objective:**

To investigate the protective effect of melatonin (MLT), as an antioxidant, on testicular damage induced by acute unilateral spermatic cord torsion and detorsion (T/D) in rats.

**Materials and Methods:**

In this experimental study, 48 adult male Wistar rats were randomly divided into three groups (8 rats/group): sham group underwent right scrotal surgery only., the T/D group underwent right testicular torsion (for 1 hr) and detorsion, and the melatonin group underwent right testicular torsion, received 25 µg/kg melatonin intraperitoneally immediately after surgery of T/D. Then the histological parameters and malondialdehyde (MDA) changes were evaluated.

**Results:**

Torsion and detorsion decreased the diameter of the tubules significantly compared to controls (p=0.003). Melatonin could increase the diameter, but it was not significant (p=0.26). The heights of the epithelium were constant in sham, T/D, and melatonin groups without any significant difference between groups (p=0.98). Based on Johnsen’s score, spermatogenesis was normal in the sham group. The torsion significantly injured all lineage cells (p<0.001). There was no any spermatid or sperm in the seminiferous tubules. Melatonin improved the spermatogenesis significantly (p=0.02), but could not improve MDA level significantly (p=0.99).

**Conclusion:**

Severe degenerative changes of testis were induced by acute unilateral spermatic cord torsion and detorsion in rats, but it had no effect on MDA level.

## Introduction

Testicular torsion because of inducing irreversible ischemic and loss of organs, appears to be an urological emergency. Its prevalence is 1:4000 in men younger than 25 yr and nearly 61% of this patients are up to 21 yr (usually with two peaks in infancy and 13 yr of age) who suffer from this problem (1). Testicular torsion, before birth or in the first 30 days after the birth, is another case that occurs less frequently (2). Delay in diagnosis and treatment leads to testicular damage and impaired fertility (3). The intensity of the damages depends on the severity of twisting and duration of ischemia (4). Treatment of testicular torsion is surgery. However, about 65% of these patients are suffering from atrophy of the testes even after surgical treatment (5). Testicular torsion decreases total antioxidants status and increases total oxidants status and oxidative stress index (6). Oxygen-free radicals are likely to result in the I/R injury (7). 

Melatonin, a hormone that is secreted by the pineal gland, is considered as a strong endogenous antioxidant. Because of its ability in scavenger of free radicals, the use of exogenous melatonin reduces oxidative stress and inflammation. Also, melatonin can increase the inherent antioxidant levels, such as glutathione peroxidase, superoxide dismutase and glutathione by an indirect mechanism (8). Melatonin, by having lipophilic and hydrophilic compounds, can pass freely from the morpho-physiological barrier of different organs (9-11). The direct effect of melatonin was observed in the male reproductive system and in the synthesis of testosterone from the Leydig cells in animals (12). In several studies, the effect of melatonin is observed on ischemia-reperfusion-induced oxidative damages in the testis (13-16). It is demonstrated that 17 mg/kg melatonin treatment 15 minutes before T/D and then melatonin treatment daily for a week could improve malondialdehyde (MDA) and increase the Johnsen's criteria (7). In another study, testicular vessels were clamped for 1 hr. Animals were received a single dose of 10 mg/kg melatonin 10 min before ischemia. MDA was not changed between the groups but abnormal sperm rate was returned to normal in the melatonin group after it has been increased in ischemia (17). 

In another study, 1 mg/kg melatonin was injected to male rats without any intervention for two months. Melatonin treated rats fertilized only 25% of the mated rats versus 87.5% in control group. The number of spermatozoa was similar in two groups but Leydig cells have shown increased level in the melatonin group. Leydig cells were like as the control group, two months after removing the treatment but ability to fertilize was the same as treatment period with a decrease in the melatonin group (18). Although several studies have examined the antioxidant effect of melatonin on testicular damage, most of these have reported different effects and there is a discrepancy between the results (7, 14, 16, 19, 20). 

Moreover, all studies about the effects of melatonin on torsion and detorsion (T/D) were performed before the trauma, whereas enough time was needed to transfer the patient to a hospital. So, we designed a study on the effect of melatonin on acute testis T/D following 1 hr after starting ischemia in a rat model. 

## Materials and methods

**Animals**

In this experimental study, 48 adult male Wistar rats weighing 180-200 gr were used. For acclimation to laboratory environments, animals were maintained in a room with constant temperature (23±2^o^C) and humidity (55-60%) with 12 hr light/dark from 1 wk before the experiment. Animals were randomly divided into three groups (8 rats/ group): Sham group, by an operation, the right testicle was elicited and then immediately returned, T/D group, underwent right spermatic cord torsion for 1 hr, and then detorsion was performed, and melatonin group, received a single dose of melatonin (25 mg/kg) just after the end of T/D. The sham and T/D groups were received normal saline the same volume as the latter group. Melatonin was dissolved in ethanol and then diluted with distilled water, therefore, we had 1% ethanol at the end.

**Creation of testicular torsion/detorsion model**

The lower region of animal’s abdomen was shaved and disinfected with betadine and alcohol. Under anesthesia by intraperitoneal injection of ketamine (5 mg/kg) and xylazine (50 mg/kg), and by an abdominal incision in the inguino-genital area, right testicle was delivered. Gubernaculum was released and spermatic cord and testicle were rotated 720 degrees in a clockwise direction. Testicle was fixed in this position for 1 hr by suturing to scrotum (14). Then, testicle was distorted and placed in the scrotum and gubernaculum was sutured (21). Rats were then housed individually in cages. 

**Collection tissue samples**

Under anesthesia, animals were sacrificed 72 hr after melatonin injection and testes were removed and fixed. For a better fixation, tunica albuginea was pierced in several points by a needle 5 hr after starting fixation. Testes were divided by a longitudinal incision into two halves 12 hr after starting fixation. Half of the animals were separated for MDA assay. The testes were transferred to -80^o^C and stored until the testing time.

**Histological evaluation**

After fixation of both testicles in 10% formalin and tissue processing, paraffin blocks were prepared. 5 µm sections were stained with hematoxylin and eosin. Five slides were prepared for each tissue. Slides were examined by a pathologist in a blind manner. The average diameter of seminiferous tubules was observed by Leitz microscope in the eyepiece 10× and objective lens 40× and measured by the Bio report OLYSIA Soft Imaging System GmbH, version 3.2 (Japan). For each slide, 10 tubules were assessed. The average thickness of the epithelium, the distance between the basement membrane to lumen seminiferous tubules with a field of 20× at angles of 90, 180, 270 and 360 degrees in 10 tubules were calculated in each slide. The severity of damage to germ cells was evaluated with Johnsen's score. 

Johnsen's score in details is as follows: Score 1: No seminiferous epithelium; Score 2: No germinal cells, Sertoli cells only; Score 3: Spermatogonia only; Score 4: No spermatozoa or spermatids, few spermatocytes; Score 5: No spermatozoa or spermatids, many spermatocytes; Score 6: No spermatozoa, no late spermatids, few early spermatids; Score 7: No spermatozoa, no late spermatids, many early spermatids; Score 8: Less than five spermatozoa per tubule, few late spermatids; Score 9: Slightly impaired spermatogenesis, many late spermatids, disorganized epithelium.; and Score 10: Full spermatogenesis (22).

**Measurement of MDA**

MDA, the end product of lipid peroxidation reacts with thiobarbituric acid and forms a red complex. For this purpose, a sample of 0.1 m was homogenized and mixed with 3 ml of 1% phosphoric acid, 0.5 ml of distilled water and 1 ml of acid 2-thiobarbituric 0.6%. The mixture was boiled in water bath for 45 min and cooled down on the ice. The thiobarbituric acid reagent was extracted by adding 4 ml of n-butanol. After centrifugation for five minutes, the optical density of 1 gr of the above mixture was measured 532 nm. 

**Ethical consideration**

The animals were maintained in standard conditions with a 12 hr light/dark cycle at constant room temperature (23±2^o^C). They had free access to water and food. All animal experiments were approved by the ethics committee of the Mazandaran University of Medical Sciences.

**Statistical analysis**

The data were analyzed with descriptive and inferential statistics. All results were analyzed by One-Way ANOVA and followed by a post-hoc Tukey test. Quantitative data of experimental and control groups were evaluated using Statistical Package for the Social Sciences, version 15.0, SPSS Inc, Chicago, Illinois, USA (SPSS) and p≤0.05 was considered as being significant. 

## Results

**Histopathology**

**Figure 1:**
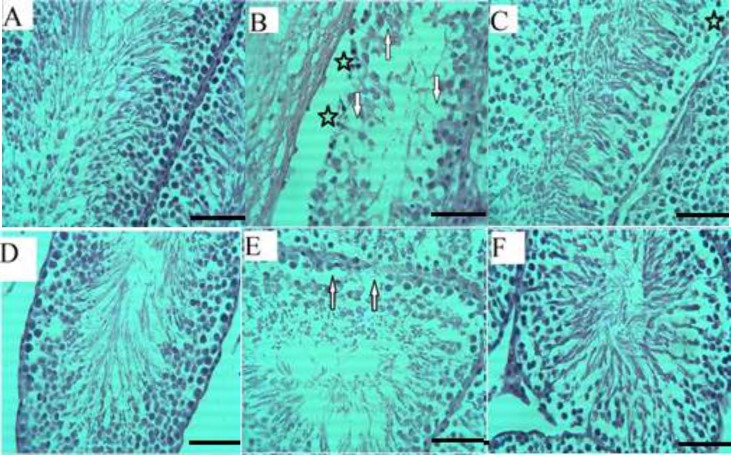
Optical microscopy of testis sections; Normal spermatogenesis is seen in the control group in both ipsilateral (A) and contralateral (D). Nearly all lineage of spermatogonial cells are damaged in torsion group. There is no any spermatid or spermatozoid in the lumen. The detachment occurred and many vacuoles are seen in ipsilateral (B) and contralateral testis (E). Melatonin could almost repair these changes. Germ cell detachments and vacuoles are diminished in melatonin group in ipsilateral (C) and contralateral testis (F). Detachment is indicated by asterisk and vacuole is showed by arrows. (H & E. ×400). Scale bar= 100 µm

In the histological evaluation, ipsilateral testes (Right) in sham group showed mild damage and contralateral testes (left) showed normal tissue structure, as shown in figure 1 (A & D). Cell layers of seminiferous tubules have preserved their normal shape, and continuity cell lines were normal and had normal spermatogenesis. Extensive degenerative changes, as well as detachment and vacuolization, have been found in T/D group, figure 1 (B & E). 

Moreover, the density of all class of cells in damaged tubules has been obviously reduced. In melatonin group, there were fewer tubules with detachment, vacuolization or some degrees of degeneration compared to the T/D group. Melatonin could partially restore these changes as shown in figure 1 (C & F). Quantitative assessment of seminiferous tubules diameter and the thickness measurement of the epithelium also confirmed the results of the histopathological examination. T/D decreased the diameter of tubules significantly (123.47±13.71) compared to sham (137.48±19.71) (p=0.003). Melatonin could repair these changes to some extent, but it was not significant (128.51±21.16) (p=0.26). 

Despite the reduction in quality and quantity of cells, the heights of the epithelium were constant in sham (29.59±5.86), T/D (27.60±5.71) and melatonin (27.51±4.79) groups and there was not a significant change between the groups (p=0.98). 

**Assessment of spermatogenesis**

**Figure 2.**
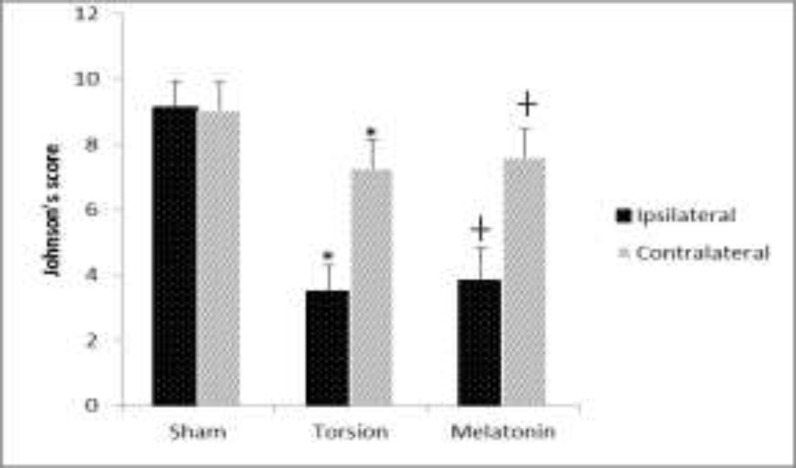
Torsion reduced all lineages of spermatogenic cells in both ipsilateral and contralateral testis by Johnsen's score. Melatonin has raised this criterion significantly. Values are shown as mean±SD. Sign (*) is indicated as significance with the sham group (p<0.001). and (┼) is indicated as significance with torsion group (p=0.02 in ipsilateral and p=0.04 in contralateral side

Based on Johnsen’s score, spermatogenesis was normal in the sham group. The torsion significantly injured all lineage cells (p<0.001). So, there was not any spermatid or sperm in the seminiferous tubules in T/D group. A few spermatogonia were observed and spermatocyte was rare in the tubules. Melatonin has improved the spermatogenesis significantly as shown in figure 2 (p=0.02). 

**MDA test**

The average MDA in the testes of both sides (the side and the opposite side) was introduced in table I. As shown in table I, in T/D group, MDA is increased significantly compared to the control group in ipsilateral testis (p=0.03). Melatonin could not improve the torsion damage (p=0.99). MDA level was not changed in contralateral testis.

**Table I:** The result of MDA levels

**Groups**	**MDA (µg/100ml)**	
	**Ipsilateral testis**	**Contralateral testis**
Sham	6.92 ± 0.27	4.06±0.41
Torsion/Detorsion	11.42 ± 3.64*	5.64± 1.47
Melatonin	11.19 ±1.20*	5.27± 1.16

Values are shown as Mean ± SD. Sign (*) is indicated as significance with the sham group. (p=0.03).

MDA; malondialdehyde

Ipsilateral testis; traumatic side testis

Contralateral testis; intact side testis

## Discussion

In this study, torsion of spermatic cord for 1 hour has extensively damaged both seminiferous tubule cells, especially the ipsilateral side. Several studies in the past indicated that torsion can injure testis and its germ cells (23-25). The spermatic cord torsion is an emergency event that is needed an invasive surgery procedure. Although, it is said that early surgical approaches lead to the salvation of testes, only 32% of testes salvage (23). A study in 1993 ascertained that minimum required time of torsion exposure to cause serious damage to the tissue is one hour (7). Contralateral testis problems resulting in injuries on one side are referred to as sympathetic orchiopathy (25). 

Although the main reason is still unknown, there is evidence that the blood-testis barrier is broken down at these events which may result in some immunological damages (25). In this study, the T/D has decreased the diameter of the tubules. The decreased seminiferous tubules diameter is an indicative of germ cell loss whereas an increased diameter may happen by fluid retention because of impaired emptying of tubules (26). Similar to this paper, previous studies showed that the same stress to testis could injure the germ cells and decrease the morphometric parameters of the tubules (26-29). The result of Johnsen's score also confirmed the reduction in germ cells. 

Melatonin was able to repair the germ cell damage by Johnsen's score in this study. Melatonin, a hormone which is synthesized by the pineal gland, is a potent antioxidant (29). Melatonin is both a direct scavenger of toxic hydroxyl radical and also an activator of the antioxidative enzymes such as glutathione peroxidase. It is said that the melatonin protects the DNA against oxidative damage in stress condition (7). Although melatonin altered the spermatogenesis, it could not change the morphometric parameters such as tubular diameter and heights of the epithelium significantly. Melatonin effect on spermatogenesis is studied in many types of research. Koksal *and his colleagues* in 2012 induced testis ischemia-reperfusion in rats by occluding testicular vessels with a vascular clamp. Animals were treated by 10 mg/kg Melatonin 10 min before ischemia. After 24 hr, samples were elicited for histopathological evaluation. The result showed that the spermatogenesis was improved significantly. This study is comparable to the present work and confirms its results. We used 25 mg/kg Melatonin and assessed morphometric parameters after 72 hr (14). 

In a study conducted by Müslim Yurtçu, 17 mg/kg Melatonin in two groups, 1 and 7 doses were injected to rats that underwent 6 hr torsion. Melatonin was injected IP 15 min before detorsion. Samples were assessed 3 months after detorsion. A single dose of Melatonin couldn’t alter The histological structure or Johnsen's score whereas 7 doses improved it (23). This is in contrary with our result. It may be due to different dosages that we used. Also, they took samples after a long time whereas we took our sample after 72 hr. It seems that a single dose of Melatonin cannot preserve its effects after a few months and more doses need to be used. 

Ghasemi *et al* in 2010 treated rats that their testes were injured by busulfan injection, 10 mg/kg Melatonin for 5 days. They showed that Melatonin could alter spermatogenesis (30). This is similar to our result. Hemadi *et al* in 2011 indicated that one week Melatonin treatment of neonate vitrified testis, that was grafted, could raise the epithelium thickness and improve the spermatogenesis (31). Conversely, heights of the epithelium were unchanged in our study. Germ cells were extensively detached from basement membrane in T/D group. The reason why could be that the total epithelium height, from the basement membrane to apical germ cells, likely contains a gap due to detachment which may cause such results. Reduction in vacuolization and detachment was obvious in the Melatonin group in optical microscopic observation. Also, we may obtain different results if we evaluate these parameters after several dosages in a longer time period. Ischemia and reperfusion are believed to increase many adverse factors containing reactive oxygen (23). 

For these reasons, many researchers have used many antioxidants such as allopurinol, polyethylene glycol-superoxide dismutase (PEG-SOD), catalase, calcium channel blockers, ginkgo biloba, sildenafil, taurine, selenium, and melatonin in induced testis torsion experimental studies (23, 32) . In this studies, increase in MDA, as a marker of oxidative stress, were seen at ipsilateral and contralateral testes in T/D group. Many oxidants may damage the biological molecules and cause irreversible oxidation reactions which can lead to cellular dysfunction (33). Torsion caused oxidation reactions and increased the MDA levels in this study. We have indicated that melatonin reduces the MDA levels and the tissue damage. This is not consistent with the results of Koksal *et al* that ischemia time was the same (14). 

The reason could be due to biochemical test which was assessed 24 hr after T/D in their experiment, but in our study, this test was performed 72 hr after melatonin treatment. Also, it may be due to the injection time and dose of melatonin, which were injected before ischemia and 10 mg/kg doses of melatonin whereas we used 25 mg/kg after detorsion.

## Conclusion

This study showed that treatment with melatonin has a protective effect on T/D-induced testicular damage containing biochemical and histopathological parameters. This result is due to the effect of melatonin on oxidative factors such as MDA. It is also shown that ipsilateral testicular damage could partially damage the contralateral testicle.
